# Rising temperature stimulates the biosynthesis of water-soluble fluorescent yellow pigments and gene expression in *Monascus ruber* CGMCC10910

**DOI:** 10.1186/s13568-017-0441-y

**Published:** 2017-06-24

**Authors:** Tao Huang, Hailing Tan, Gong Chen, Lu Wang, Zhenqiang Wu

**Affiliations:** School of Bioscience and Bioengineering, Guangdong Provincial Key Laboratory of Fermentation and Enzyme Engineering, South China University of Technology, Guangzhou, 510006 People’s Republic of China

**Keywords:** *Monascus ruber*, Water-soluble yellow pigments, Strong fluorescence, Gene regulation, Temperature-associated

## Abstract

*Monascus* species can produce secondary metabolites that have a polyketide structure. In this study, four types of extracellular water-soluble yellow pigments (**Y1**–**Y4**) were generated by submerged fermentation with *Monascus ruber* CGMCC 10910, of which **Y3** and **Y4** had strong yellow fluorescence. The composition of the pigment mixtures was closely related to the fermentation temperature. The dominating pigments changed from **Y1** to **Y3** and **Y4** when fermentation temperature increased from 30 to 35 °C. Increasing the temperature to 35 °C changed the metabolic pathways of the pigments, which inhibited the biosynthesis of **Y1** and enhanced the biosynthesis of **Y3** and **Y4**. Moreover, the yield of **Y1** reduced insignificantly, while the yields of **Y3** and **Y4** increased by 98.21 and 79.31% respectively under two-stage temperature fermentation condition. The expression levels of the relative pigment biosynthetic genes, such as *MpFasA2*, *MpFasB2*, *MpPKS5*, *mppR1*, *mppB*, and *mppE,* were up-regulated at 35 °C. The two-stage temperature strategy is a potential method for producing water-soluble *Monascus* yellow pigments with strong yellow fluorescence.

**Electronic supplementary material:**

The online version of this article (doi:10.1186/s13568-017-0441-y) contains supplementary material, which is available to authorized users.

## Introduction

*Monascus* spp. has been widely used in the production of *Monascus* pigments for colouring traditional foods in Asian centuries (Juzlova et al. 1996). Many of the identified *Monascus* pigments are fungal metabolites called azaphilones with a polyketide structure, including the six well-known *Monascus* pigments red (monascorubramine and rubropunctamine), orange (monascorubrin and rubropunctatin), and yellow (monascin and ankaflavin) (Patakova 2013). Some azaphilone metabolites from *Monascus* spp. are known to have fluorescent characteristics (Table 1).

**Table 1:** Summary of the reported *Monascus* pigments with fluorescence

MPs compound name	Molecular formula	λ ex, λ em (nm)	Pigments colour	Fluorescent colour	References
Monasfluore A	C_21_H_24_O_5_	396, 460	Yellow	Blue	Huang et al. (2008)
Monasfluore B	C_23_H_28_O_5_	396, 460	Yellow	Blue	Huang et al. (2008)
Monarubrin	C_20_H_26_O_4_	340, 490	Yellow	Blue	Loret & Morel (2010)
Rubropunctin	C_22_H_30_O_4_	340, 490	Yellow	Blue	Loret & Morel (2010)
Monaphilol A	C_23_H_29_O_5_	438, 554	Orange	Yellow	Hsu et al. (2011)
Monaphilol B	C_21_H_25_O_5_	438, 554	Orange	Yellow	Hsu et al. (2011)
Monaphilol C	C_26_H_33_O_6_	480, 540	Orange	Yellow	Hsu et al. (2011)
Monaphilol D	C_24_H_29_O_6_	480, 540	Orange	Yellow	Hsu et al. (2011)
MMA	C_22_H_26_O_6_	472, 535	Orange	Yellow	Huang et al. (2014)
MMB	C_24_H_30_O_6_	472, 535	Orange	Yellow	Huang et al. (2014)

The medium composition in submerged fermentation has a significant influence on the yield and quality of the *Monascus* yellow pigments (Chen and Johns 1994; Yongsmith et al. 1993). Similarly, cultivation conditions also affect the production of *Monascus* yellow pigments, for example, low pH facilitates the production of yellow pigments (Shi et al. 2015). Suitable blue light can stimulate the production of yellow pigments (Chen et al. 2016). Moreover, transferring pigments from the intracellular into the extracellular environment can intensify the accumulation of yellow pigments by extractive fermentation in a nonionic surfactant micelle aqueous solution (Kang et al. 2013; Xiong et al. 2015). Temperature is a critical environmental signal for regulating the developmental and physiological processes in microorganisms. Low temperatures (24 or 25 °C) yield higher amounts of the pigment during the submerged fermentation of *Penicillium purpurogenum* GH2 or *Monascus* sp. J101 (Mendez et al. 2011; Ahn et al. 2006). High temperature (>45 °C) could also induce the production of yellow pigments by *Monascus purpureus* LPB 97 in a solid culture (Babitha et al. 2007). A gene cluster for the biosynthetic azaphilone pigments as well as functions of the critical genes that were involved in the biosynthetic pathway was reported in the genome of *Monascus pilosus* (Balakrishnan et al. 2013).

Most *Monascus* yellow and red pigments reported are intracellular alcohol-soluble pigments combined with a few extracellular water-soluble pigments (Feng et al. 2012; Chen and Wu 2016). Many studies focused on the fermentation of intracellular yellow pigments (Xiong et al. 2015; Chen and Wu 2016; Krairak et al. 2000), but not the extracellular pigments, especially on the water-soluble ones. Water-soluble yellow pigments can be synthesized via chemical modification of intracellular alcohol-soluble pigments (Edward et al. 1991), which had a potential risk in food application. Recently, we found that the extracellular water-soluble yellow pigments could be efficiently produced by *M. ruber* CGMCC 10910 under high glucose concentration with low oxidoreduction potential (ORP) (Wang et al. 2017). In this study, we will investigate the effect of temperature on the biosynthesis of the water-soluble yellow pigments and their correlation with the expression levels of relative genes in *M. ruber* CGMCC 10910.

## Materials and methods

### Microorganisms and media

*Monascus ruber* CGMCC 10910 (China General Microbiological Culture Collection Center, CGMCC 10910) used in this study was cultivated on PDA medium at 30 °C for 7 days and stored at 4 °C. The inoculum culture medium contained (g/L): glucose, 20; yeast extract, 3; peptone, 10; KH_2_PO_4_, 4; KCl, 0.5; and FeSO_4_·7H_2_O, 0.01. The fermentation culture medium contained (g/L): glucose, 150; (NH_4_)_2_SO_4_, 5; KH_2_PO_4_, 5; MgSO_4_·7H_2_O, 0.5; KCl, 0.5; MnSO_4_·H_2_O, 0.03; ZnSO_4_·7H_2_O, 0.01; and FeSO_4_·7H_2_O, 0.01.

### Cultivation

The strain was grown on PDA plates at 30 °C for 7 days. Single colonies (5–6 loops) of approximately 10 mm diameter were scraped off the agar plate and used to inoculate 50 mL of the inoculums culture medium in a 250-mL Erlenmeyer flask and grown at 30 °C and 180 rpm for 25 h. Then, an 8% (v/v) inoculum culture was inoculated into a 25 mL fermentation culture medium in a 250-mL Erlenmeyer flask and cultured at 30 °C and 180 rpm for 8 days. Different temperature cultures were grown in a constant temperature shaker set at 25, 30 and 35 °C for 8 days. A two-stage culture was maintained in a constant temperature shaker as follows: A, cultured at 30 °C for the first 6 days and 35 °C for the last 2 days; B, cultured at 30 °C for the first 4 days and 35 °C for the last 4 days; C, cultured at 35 °C for the first 4 days and 30 °C for the last 4 days; D, cultured at 35 °C for the first 6 days and 30 °C for the last 2 days. All experiments were performed in triplicates.

### Determination of DCW and glucose concentration

The fermentation broth after culture was filtered through a 0.8 mm mixed cellulose esters membrane. The filtrate (extracellular broth) was appropriately diluted to determine the residual glucose concentration. The residual glucose was quantified by the standard 3,5-dinitrosalicylic acid (DNS) method. The mycelia were filtered from the culture broth, subjected to washing 3 times with distilled water and dried at 60 °C in the oven for at least overnight until a constant weight was achieved for the determination of dry cell weight (DCW) by gravity.

### Pigments analysis with spectrophotometer, TLC, HPLC and LC–MS

The filtrate (extracellular broth) was appropriately diluted to determine the concentration of the extracellular pigment. The absorbance spectrum of the extracellular pigments was recorded by a UV–Visible spectrophotometer (Unico, USA) from 300 to 550 nm at 1 nm interval.

The concentration of the intracellular pigments was determined by the following procedure: the harvested washed mycelia were re-suspended in a 25 mL acidic aqueous ethanol (70% v/v pH = 2 with hydrochloric acid) and incubated for 1 h. The suspension was filtered with filter paper and the filtrate (intracellular extract) was appropriately diluted to determine the concentrations of the intracellular pigments. The absorbance spectrum of the intracellular pigments was recorded by a UV–Visible spectrophotometer (Unico, USA) from 300 to 550 nm at 1 nm intervals. Moreover, the absorbance units (AU) at peak wavelengths of 410 nm multiplied by the dilution ratio were used as indexes for the intracellular yellow pigments concentrations (Shi et al. 2015).

Thin-layer chromatography analysis was conducted on a silica gel 60 F_254_ TLC plate (Merck, Germany) with chloroform/methanol/acetic acid (285:21:9) as the mobile phase (Xiong et al. 2015), the amount of sample loaded on the TLC plate was approximately 10 µL. The spots on TLC were detected under ultraviolet (UV) lamps (356 nm).

HPLC-PDAD and HPLC-FLD analysis extracellular pigments were performed on an Alliance e2695 system (Waters, Milford, CT, USA) equipped with a 2998 photodiode array (PDA) detector (Waters, Milford, CT, USA) and a 2475 multi-wavelength fluorescence (FLD) detector (Waters, Milford, CT, USA) using a Zorbax Eclipse Plus C18 column (250 × 4.6 mm, 5 μm, Agilent, Palo Alto, CA, USA). The column temperature was set at 30 °C. Mobile phase A (aqueous H_3_PO_4_, pH 2.5) and mobile phase B (acetonitrile) were used according to the following gradient at 0.8 mL/min flow rate: 0 min, 90% A, 10% B; 15 min, 80% A, 20% B; 20 min, 80% A, 20% B; 21 min, 20% A, 80% B; 31 min, 20% A, 80% B; 32 min, 90% A, 10% B; 40 min, 90% A, 10% B.

Liquid chromatography–mass spectrometry consisted of an HP1100 HPLC system (Agilent, Palo Alto, CA, USA) and a microTOF-QII mass spectrometer (Bruker, Rheinstetten, Germany). The C18 column and chromatographic conditions were the same as mentioned above, except for the mobile phase A was water with 0.1% formic acid.

### Gene analysis with real-time quantitative PCR

The effects of the fermentation temperature on the expression of key genes during pigments production were investigated using real-time quantitative PCR. The mycelia were collected for total RNA extraction using the Plant RNA Extraction Kit (TakaRa MiniBEST). cDNA was synthesized using the PrimeScript™RT reagent Kit with gDNA Eraser (TaKaRa). Primers for the amplification of *MpFasA2*, *MpFasB2*, *MpPKS5*, *mppR1*, *mppA, mppB*, *mppC*, *mppD*, *mppE*, *mppR2* (GenBank Accession No. KC148521) and the *actin* gene (GenBank Accession No. AJ417880) were used according to Wang et al. (2015) with modifications (Additional file 1: Table S1). The *actin* gene was used as the reference gene. Gene expression was monitored by RT-qPCR using the SYBR Premix Ex TaqII (TaKaRa). RT-qPCR was performed using a Lightcycler 96 (Roche, USA) with the following cycling program: pre-incubation at 95 °C for 30 s, followed by a two-step amplification (40 cycles of denaturation at 95 °C for 5 s, and annealing at 60 °C for 30 s) and dissociation curve analyses (at 95 °C for 10 s, annealing at 65 °C for 60 s, and plotting dissociation curves from 65  to 95 °C, with a final incubation at 97 °C for 1 s).

### Statistical analysis

Each experiment was repeated at least thrice. Numerical data are presented as the mean ± SD. The differences among different treatments were analyzed using one-way ANOVA. All statistical analyses were performed by using the SPSS 22.0, software; *p* < 0.05 and *p* < 0.01 were considered statistically significant.

## Results

### Pigment biosysthesis in *M. ruber* CGMCC 10910 under different temperatures

**Fig. 1:**
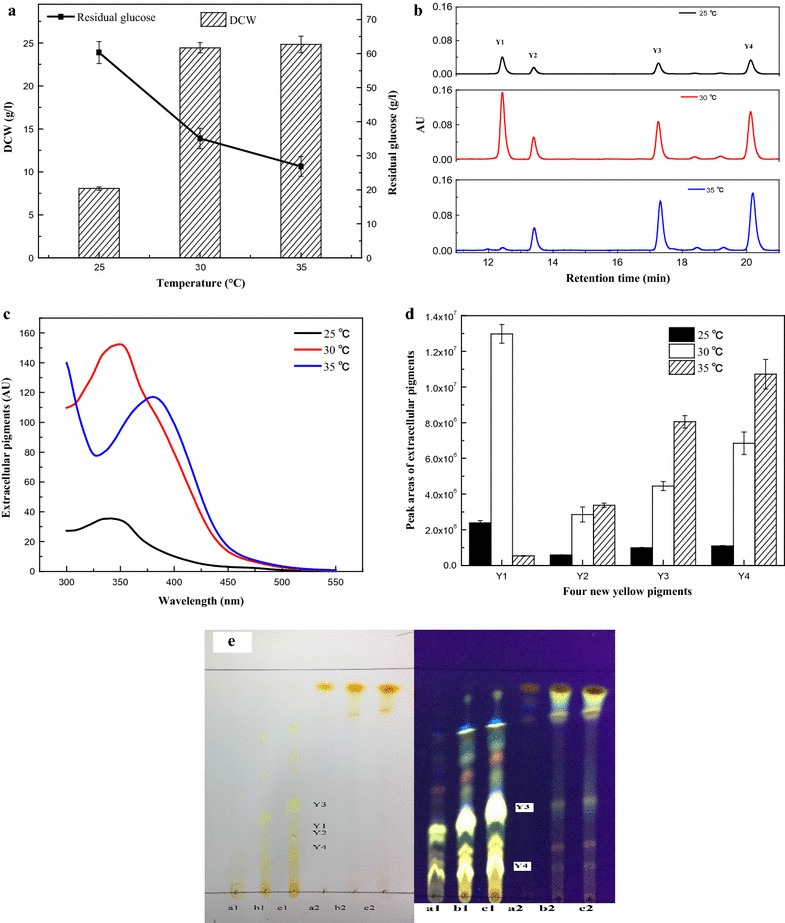
Cell growth and pigment production in submerged fermentation of *M. ruber* CGMCC 10910 at 25, 30, and 35 °C. **a** Residual glucose (g/L) and DCW (g/L). **b** The profile of extracellular pigments determined by HPLC-PDAD at 388 nm. **c** UV–Visible spectra of extracellular pigments. **d** Yields of extracellular four water-soluble yellow pigments. **e** TLC analysis of the extracellular and intracellular pigments, the left one is in visible light, the right one is in UV lamp (365 nm). “a1, b1, c1”, respectively, represent extracellular pigments of fermentation temperature at 25, 30, and 35 °C, and “a2, b2, c2” represent intracellular pigments

**Fig. 2:**
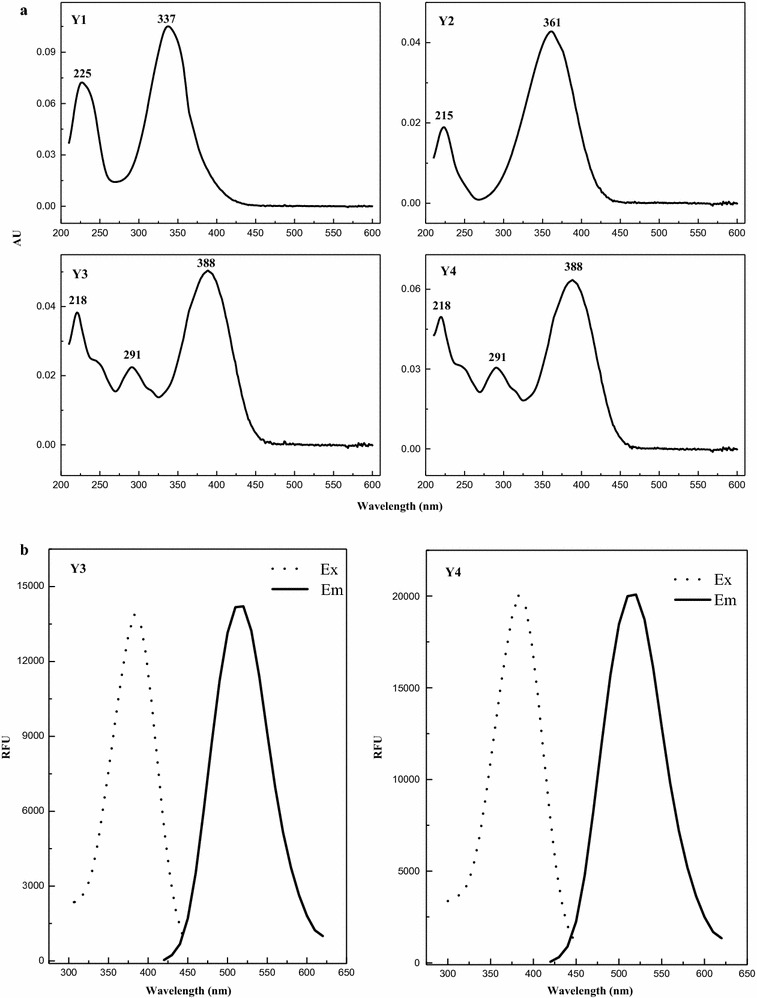
**a** UV–Visible spectra of extracellular water-soluble yellow pigments detected by HPLC-PDAD. **Y1**, retention time at approximately 12.3 min; **Y2**, retention time at approximately 13.3 min; **Y3**, retention time at approximately 17.2 min; **Y4**, retention time at approximately 20.1 min. **b** Excitation and emission spectra of **Y3** and **Y4** detected by HPLC-FLD, *λ*ex = 388 nm, *λ*em = 520 nm

Fermentation temperature of 30 and 35 °C were suitable for both cell growth and glucose consumption; however, a low temperature of 25 °C was not suitable (Fig. 1a). The extracellular pigments were mixtures of four components, including **Y1**, **Y2**, **Y3** and **Y4** (Fig. 1b). The UV–Visible spectra absorption peak of the extracellular pigment compositions was at 350 nm at 25 and 30 °C, and 388 nm at 35 °C (Fig. 1c). The HPLC profile (Fig. 1b) and peak areas (Fig. 1d) of extracellular pigment compositions showed that their biosynthesis at 25 °C was strongly inhibited, whereas it increased significantly at 30 and 35 °C. The relative proportion of the four pigment components was almost the same at 25 and 30 °C, and **Y1**, **Y3** and **Y4** were the dominant pigments. However, **Y3** and **Y4** were dominated pigments at a higher temperature of 35 °C, and **Y1** was almost undetectable (Fig. 1b, d). The proportion of **Y2** did not vary significantly at 30 and 35 °C. Moreover, **Y1** was in the UV–Visible spectrum with two maximum absorptions at approximately 225 nm and 337 nm, **Y2** showed the UV–Visible spectra with two maximum absorption at approximately 215 and 361 nm, **Y3** and **Y4** showed similar UV–Visible spectra with three maximum absorptions at approximately 218, 291 and 388 nm (Fig. 2a). This could explain the change in the absorption peak (from 350 to 388 nm) of extracellular pigments in response temperature shift. The TLC results showed that all samples from 25, 30, and 35 °C temperature treatments had four yellow color spots (**Y1**–**Y4**) under visible light, including two spots (**Y3**, **Y4**) with strong yellow fluorescence under ultraviolet lamp (365 nm) (Fig. 1e). Moreover, the sample at 35 °C exhibited the strongest fluorescence (Fig. 1e). **Y3** and **Y4** had almost the same UV–Visible spectra (Fig. 2a) and both exhibited the same fluorescence spectra with maximum excitation and emission at 388 nm and 520 nm, respectively (Fig. 2b). The total peak areas of **Y3** and **Y4** at 35 °C was 1.88 × 10^7^, and 7.48 × 10^6^ higher that at 30 °C, but that of **Y1** decreased to approximately 1.24 × 10^7^ (Fig. 1d). These pigments were further identified by LC–MS, which determined the molecular weights of **Y1**, **Y2**, **Y3** and **Y4** as 250, 254, 402 and 358, respectively (Table 2). **Y1** was the reported intermediate azanigerone E (C_13_H_14_O_5_) according to its UV–Visible spectra and mass spectra (Fig. 2a) (Zabala et al. 2012; Huang et al. 2017; Chen et al. 2017).

**Table 2:** Chemical characteristics of water-soluble yellow pigments produced by *M. ruber* CGMCC *10910*

No.	Peak positions of spectra (nm)^a^	λ ex, λ em (nm)	Molecular weight^b^	Pigments
Y1	225, 337	–	250.0936	Azanigerone E
Y2	215, 361	–	254.1252	Not reported
Y3	218, 291, 388	388, 520	402.1440	Not reported
Y4	218, 291, 388	388, 520	358.1171	Not reported

^a^Detected by HPLC-PDAD

^b^Detected by LC–MS

The intracellular pigments are mainly composed of two yellow pigments (monascin and ankaflavin) and two orange pigments (monascorubrin and rubropunctation) (Additional file 2: Figure S1), as demonstrated by our previous study (Huang et al. 2017). The yield of intracellular pigments was also strongly inhibited at 25 °C. Optimal temperature for the production of intracellular yellow pigments was 35 °C (Additional file 3: Figure S2), of which the final yield of the intracellular yellow pigments increased approximately 1.8 times of that at 25 °C, reaching up to 190 AU_410_.

### Conversion between pigment components in response to temperature shifts

**Fig. 3:**
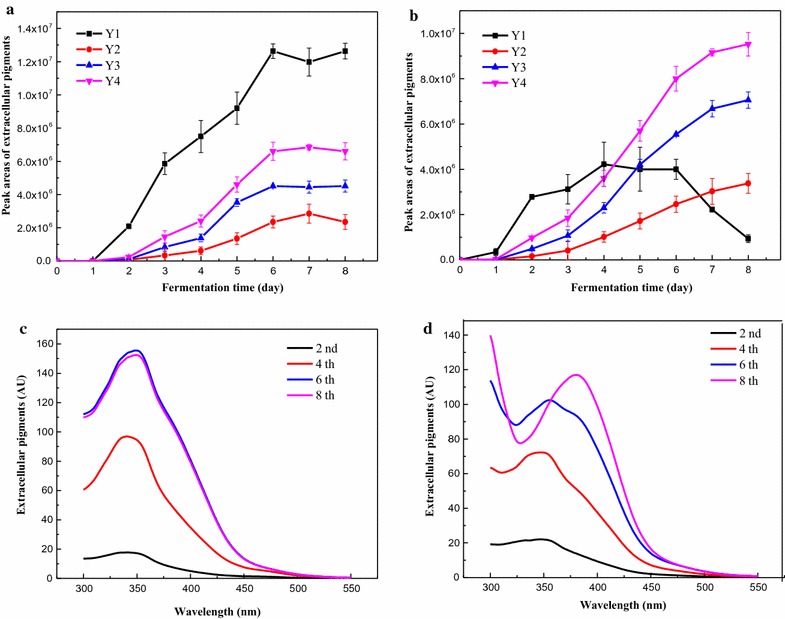
Metabolism rules of the four water-soluble yellow pigments under different fermentation temperatures. **a** and **b** Peak areas of the four water-soluble yellow pigments. **c** and **d** UV–Visible spectra of extracellular pigments on 2nd, 4th, 6th and 8th day. **a** and **c** 30 °C. **b** and **d** 35 °C

**Y1** was preferentially synthesized on the 1st day before the production of the other pigments had commenced (Fig. 3a, b), suggesting that **Y1** biosynthesis occurred before other pigments. Thereafter, the four pigments (**Y1**, **Y2**, **Y3** and **Y4**) increased rapidly and reached their maximum concentrations on the 6th day after which they remained almost constant when the fermentation temperature was 30 °C (Fig. 3a). The yield of **Y1** was much higher than the other 3 pigments, and the maximum absorption wavelength of extracellular pigments was maintained as approximately 350 nm in the whole fermentation process (Fig. 3c). However, the increase in fermentation temperature to 35 °C resulted in an increase in **Y3** and **Y4,** which increased continuously from the 2nd to the 8th day, whereas **Y1** began to decrease after a rapid increase from the 2nd to the 4th day and resulted in a very low yield on the 8th day. Thus, **Y3** and **Y4** were the dominant pigments, while **Y2** showed no obvious changes (Fig. 3b). Therefore, the maximum absorption wavelength of the extracellular pigments was shifted from 350 nm during the first 4 days to 388 nm on 8th day (Fig. 3d). We deduced that 30 °C was beneficial for the biosynthesis of **Y1**. Higher temperature at 35 °C inhibited the biosynthesis of **Y1** but was beneficial to the biosynthesis of **Y3** and **Y4**.

### Two-stage temperature strategy to improve the production of strong fluorescent water-soluble yellow pigments

**Fig. 4:**
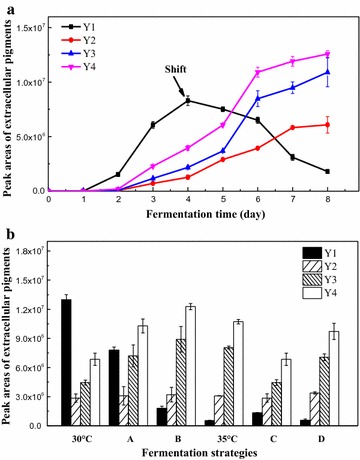
Temperature-shift strategy fermentation on the production of extracellular four water-soluble yellow pigments. **a** Time course of the four new water-soluble yellow pigments fermentation under B strategy. **b** Yields of the four water-soluble yellow pigments under different fermentation strategies. Fermentation strategies: 30 and 35 °C respectively represented fermentation at 30 and 35 °C for 8 days; *A* 30 °C for 6 days and then turn into 35 °C for 2 days; *B* 30 °C for 4 days and then turn into 35 °C for 4 days; *C* 35 °C for 4 days and then turn into 30 °C for 4 days; *D* 35 °C for 6 days and then turn into 35 °C for 2 days

Our results showed that a temperature of 30 °C was beneficial for the accumulation of **Y1** while 35 °C inhibited its production and improved the production of **Y3** and **Y4** (Fig. 3). The growth rate of **Y1** began to decrease on the 2nd day, and the yield started to decline from the 4th day towards when culture temperature was 35 °C; however, **Y2**, **Y3** and **Y4** increased continuously until the 8th day. Based on these results, we designed a two-stage temperature control fermentation experiment. The culture temperature in this experiment was maintained at 30 °C for the first 4 days, and then increased to 35 °C for the last 4 days. The time course of this two-stage temperature control fermentation experiment showed that at 35 °C, **Y1** decreased in the later stage of culture reaching a significantly low level, while the peak areas of **Y2**, **Y3** and **Y4** increased continuously until the 8th day (Fig. 4a). Finally, the absorption peak of the extracellular pigments increased to 388 nm and **Y3** and **Y4** become the dominant pigments.

To choose a suitable strategy for temperature-shifting fermentation, we changed the culture temperature from 30 to 35 °C or from 35 to 30 °C at different fermentation times. Consequently the yields of **Y3** and **Y4** were higher than that of the constant temperature culture (at 30 and 35 °C) during the temperature shift from 30 to 35 °C. However, the shift from 35 to 30 °C did not increase and even decreased in some degree (Fig. 4b). The two-stage temperature control strategy evidently caused the yield of yellow fluorescent pigments (**Y3** and **Y4**). Moreover, the temperature shift from 30 to 35 °C was better for the accumulation of yellow fluorescent pigments (**Y3** and **Y4**) compared with a constant temperature culture. The shift on the 4th day was the best for the production of **Y3** and **Y4**, wherein the yield of **Y3** and **Y4** improved by 98.21 and 79.31% compared with constant temperature (at 30 °C) fermentation (Fig. 4b).

### Gene expression for the biosynthesis of pigments under different temperatures

**Fig. 5:**
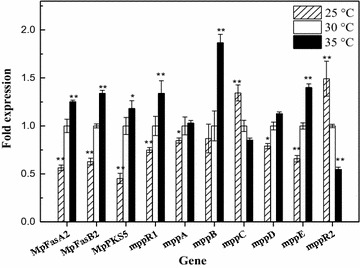
Relative expression levels of the pigment biosynthesis related genes *MpFasA2, MpFasB2, MpPKS5, mppR1, mppA, mppB, mppC, mppD, mppE* and *mppR2* as monitored by qRT-PCR. *Error bars* represent the standard deviation (n = 3). **p < 0.01; *p < 0.05

The relative expression levels of the pigment biosynthetic genes *MpFasA2, MpFasB2, MpPKS5, mppR1, mppA, mppB, mppC, mppD, mppE* and *mppR2* were monitored under different fermentation temperatures using RT-qPCR (Fig. 5). The test samples used to study gene expression were the same samples used for pigment testing. Transcription levels were normalized to that of the *actin* gene and the control (fermentation at 30 °C) as the reference value (value 1). The expression levels of the genes *MpFasA2, MpFasB2, MpPKS5, mppR1, mppB,* and *mppE* at a low temperature (25 °C), were significantly down-regulated (*p* < 0.01 or *p* < 0.05). Moreover, the down-regulated expression of these genes was positively correlated with the production of intracellular and extracellular pigments (Fig. 1). However, the genes *mppC* and *mppR2* that were negatively correlated with pigment production were significantly up-regulated (*p* < 0.01). However, a higher temperature (35 °C) significantly up-regulated the levels of genes expression for *MpFasA2, MpFasB2, MpPKS5, mppR1,* and *mppE,* especially *mppB* (encodes a trichothecene 3-*O*-acetyltransferase) (*p* < 0.01 or *p* < 0.05). Moreover, the expression of *mppR2* (Balakrishnan et al. 2013) was significantly down-regulated (*p* < 0.01) as well as that of the *mppC*. It was demonstrated that temperature inhibition (fermentation at 25 °C) or stimulation (fermentation at 35 °C) the production of pigments through down-regulating or up-regulating the relative expression levels of *MpFasA2, MpFasB2, MpPKS5, mppR1, mppB,* and *mppE* (Figs. 1, 5).

## Discussion

Four types of extracellular water-soluble yellow pigments (**Y1**–**Y4**) were found in the fermentation broth with *M. ruber* CGMCC 10910, two (**Y3** and **Y4**) of which showed strong yellow fluorescence. Based on their UV–Visible spectra, fluorescence spectra and mass spectra, **Y1** was the reported intermediate azanigerone E (Zabala et al. 2012; Chen et al. 2017), and we concluded that the other compounds (**Y2**–**Y4**) were novel and had not been previously reported (Table 1; Fig. 2). The molecular weights of **Y3** and **Y4** were 402 and 358, respectively (Table 2). They had similar UV–Visible spectra and fluorescence spectra, indicating that they had the same chromophore (Huang et al. 2008). Moreover, **Y1** biosynthesis occurred before other pigments (Fig. 3), and the decrease of **Y1** followed the increase in **Y3** and **Y4** (Fig. 3). The azaphilone compound was reported with a low molecular weight for lacking the acyl moiety on the side chain. It generally acts as an intermediate for pigment biosynthesis, such as M7PKS-1 (C_13_H_16_O_5_) (Liu et al. 2016), azanigerone E (C_13_H_14_O_5_) (Zabala et al. 2012; Chen et al. 2017). We speculated that **Y1** maybe act as an intermediate of other pigments and could be converted into **Y3** and **Y4** at suitable fermentation temperatures.

Temperature regulates secondary metabolite production via up- or down-regulating the expression levels of the relative genes (Liao et al. 2009). In this study, culture temperatures of 30 °C and 35 °C were beneficial to *M. ruber* cell growth, corroborating to the reported that the optimum temperature of different *Monascus* strains was between 30 and 37 °C (Domsch et al. 1980). A lower temperature inhibited the pigment production while a higher temperature (35 °C) was more suitable for pigment biosynthesis (Fig. 1). Analysis of the relative expression levels of the pigment biosynthetic genes showed that the structural genes *MpFasA2, MpFasB2, MpPKS5, mppE,* and *mppB,* and the regulatory gene *mppR1* (Balakrishnan et al. 2013) were positively correlated with the production of intracellular and extracellular pigments, whereas *mppC* and *mppR2* showed a negative correlation (Fig. 5). Interestingly, the UV–Visible spectra of the extracellular broth strongly were influenced by the culture temperature in submerged fermentation by *M. ruber* CGMCC 10910 (Fig. 1c). HPLC profiles showed that the composition of the four water-soluble yellow pigments was different at a higher temperature (35 °C) compared with cultures grown at 30 °C, wherein the dominated water-soluble yellow pigments changed from **Y1** to **Y3** and **Y4** (Fig. 1). The culture conditions and the method usually shifted the pigment characteristics and productivities (Chen et al. 2015). The time course study of cultures at 35 °C showed that at a later stage (from the 4th day) **Y1** decreased and **Y3** and **Y4** increased continuously, resulting in higher yields of **Y3** and **Y4** but a lower yield of **Y1** (Fig. 3). *Monascus* pigments are sensitive to pH, UV and temperature (Mapari et al. 2009). The thermostability experiment confirmed that **Y1** was very stable under a higher temperature (35 °C) (Additional file 4: Figure S3). Therefore, the decrease of **Y1** content could be caused by the transformation into other pigments (**Y3** and **Y4**) rather than self-degradation at 35 °C.

Temperature-shifting cultivation has been applied as a simple technology to improve the production in submerged cultures (Ansorge and Kula 2000; Zhou and Kang 2012; Polburee et al. 2016). In this study, a temperature-shifting cultivation strategy was applied to the production of extracellular water-soluble yellow pigments. Our results showed that the culture temperature shift from 30 to 35 °C in the middle of the culture course was better for the accumulation of yellow fluorescent pigments (**Y3** and **Y4**) compare with constant temperature cultures (Fig. 4b). Exposure to elevated temperatures instigates, many organisms to rapidly synthesize a highly conserved set of proteins termed as heat shock proteins (or heat shock factor) and their induction is putatively correlated to the adaptation of the organism to hypothermic stress conditions (Schlesinger 1990). Heat shock factor σ^H^ could activate the biosynthesis of streptomycin by *Streptomyces griseus* when exposed to higher temperatures (Horinouchi 2002). Thus, there may be some heat shock proteins (or heat shock factors) associated with the synthesis of extracellular water-soluble yellow pigments synthesis (**Y3** and **Y4**), which enhanced the expression of relative genes at elevated temperatures and facilitated the biosynthesis of **Y3** and **Y4**. The temperature shifted from 30 to 35 °C on the 4th day was an effective way to improve the production of **Y3** and **Y4** (Fig. 4b).

In conclusion, *M. ruber* CGMCC 10910 produced four types of water-soluble yellow pigments **Y1**–**Y4,** and their biosynthesis was temperature-associated. The biosynthesis of **Y1** was inhibited whereas biosynthesis of **Y3** and **Y4** were enhanced under higher temperatures. The two-stage temperature control strategy provides a suitable method for producing water-soluble yellow pigments with strong yellow fluorescence in submerged fermentation.

## Additional files

**Additional file 1: Table S1.** Primers for RT-qPCR analyzing pigments biosynthetic genes.

**Additional file 2: Figure S1.** The profile of intracellular pigments determined by HPLC-PDAD at 388 nm. 1, Monascin, retention time at approximately 26.7 min; 2, Ankaflavin, retention time at approximately 30.7 min; 3, Rubropunctation, retention time at approximately 26.9 min; 4, Monascorubrin, retention time at approximately 31.6 min.

**Additional file 3: Figure S2.** UV–Visible spectra of intracellular pigments.

**Additional file 4: Figure S3.** The thermostability of the four water-soluble yellow pigments in broth at 35 °C. 0 day was the broth obtained from culture at 30 °C for 8 days. 4 days and 8 days represented the incubation time of the same broth in 35 °C and shaken at 180 rpm for 4 and 8 days, respectively.

## Abbreviations

DCW: dry cell weight
HPLC: high performance liquid chromatography
PDAD: photodiode array detector
FLD: fluorescence detector
TLC: thin-layer chromatography
LC–MS: liquid chromatograph–mass spectrometer
RT-qPCR: real-time quantitative PCR
