# Supplementary material for: Rising temperature stimulates the biosynthesis of water-soluble fluorescent yellow pigments and gene expression in Monascus ruber CGMCC10910
Source: AMB Express. 2017 Jun 24;7:134. doi: 10.1186/s13568-017-0441-y (PMC5483225; doi:10.1186/s13568-017-0441-y)
Supplement: Supplementary file 1 — Additional file 1: Table S1. Primers for RT-qPCR analyzing pigments biosynthetic genes. [file 13568_2017_441_MOESM1_ESM.doc]

**Additional Table 1**

**Table S1.** Primers for RT-qPCR analyzing pigments biosynthetic genes

| Primers name | Primers sequences (5′→3′) |
| --- | --- |
| *actin* F | TTCGAGACCTTCAACGCCC |
| *actin* R | ACCCTCGTAGATGGGAACGA |
| *MpPKS5*F | TGTCCGACGAGTTTCTGCAA |
| *MPPKS5*R | TATCAACGCTGCTTGGGCAT |
| *mppR1*F | TCTGCAGTATGCCATGTGGG |
| *mppR1*R | ATGGCACCGTCACTTAGCTC |
| *mppA F* | GCTGATTTCGGGTGTTTC |
| *mppA R* | GCTTGTTACTTTTGCTGTTC |
| *mppB* F | CGTCTCGCCCGATAACTTCA |
| *mppB* R | TTGACAGACGGGTCGAAGTC |
| *mppC* F | CAGTCCTCGTCCCTTCCAGT |
| *mppC* R | CCACGGTGAAGGATGTCGAG |
| *mppD* F | TCAACACGGGAGATGCTGTC |
| *mppD* R | GCCAAAGGACAGGAGCAGAT |
| *mppE* F | CTTCCCGATGCCGTTGTGAT |
| *mppE* R | CGTCTCGTGGATCATCTCGT |
| *mppR2*F | ACGAAACCCTCCATGACACC |
| *mppR2*R | TGCAGACAGCCTTGTGGTAG |
| *MpFasA2* F | ATGGATCGCCCGATCTTGTC |
| *MpFasA2* R | CTTTGTCGAGTCCGCTGGAT |
| *MpFasB2* F | CCTCCAGGGATTACAACCCG |
| *MpFasB2* R | ATTCAATGCCAGGTGCTCCA |
