# Supplementary material for: Rising temperature stimulates the biosynthesis of water-soluble fluorescent yellow pigments and gene expression in Monascus ruber CGMCC10910
Source: AMB Express. 2017 Jun 24;7:134. doi: 10.1186/s13568-017-0441-y (PMC5483225; doi:10.1186/s13568-017-0441-y)
Supplement: Supplementary file 2 — Additional file 2: Figure S1. The profile of intracellular pigments determined by HPLC-PDAD at 388 nm. 1, Monascin, retention time at approximately 26.7 min; 2, Ankaflavin, retention time at approximately 30.7 min; 3, Rubropunctation, retention time at approximately 26.9 min; 4, Monascorubrin, retention time at approximately 31.6 min. [file 13568_2017_441_MOESM2_ESM.doc]

**Additional Figure S1**

**Figure S1** The profile of intracellular pigments determined by HPLC-PDAD at 388 nm. 1, Monascin, retention time at approximately 26.7 min; 2, Ankaflavin, retention time at approximately 30.7 min; 3, Rubropunctation, retention time at approximately 26.9 min; 4, Monascorubrin, retention time at approximately 31.6min.
