# Supplementary material for: Rising temperature stimulates the biosynthesis of water-soluble fluorescent yellow pigments and gene expression in Monascus ruber CGMCC10910
Source: AMB Express. 2017 Jun 24;7:134. doi: 10.1186/s13568-017-0441-y (PMC5483225; doi:10.1186/s13568-017-0441-y)
Supplement: Supplementary file 3 — Additional file 3: Figure S2. UV–Visible spectra of intracellular pigments. [file 13568_2017_441_MOESM3_ESM.doc]

**Additional Figure S2**

**Figure S2**  UV-Visible spectra of intracellular pigments.
