# Supplementary material for: Rising temperature stimulates the biosynthesis of water-soluble fluorescent yellow pigments and gene expression in Monascus ruber CGMCC10910
Source: AMB Express. 2017 Jun 24;7:134. doi: 10.1186/s13568-017-0441-y (PMC5483225; doi:10.1186/s13568-017-0441-y)
Supplement: Supplementary file 4 — Additional file 4: Figure S3. The thermostability of the four water-soluble yellow pigments in broth at 35 °C. 0 day was the broth obtained from culture at 30 °C for 8 days. 4 days and 8 days represented the incubation time of the same broth in 35 °C and shaken at 180 rpm for 4 and 8 days, respectively. [file 13568_2017_441_MOESM4_ESM.doc]

**Additional Figure S3**

**Figure S3** The thermostability of the four water-soluble yellow pigments in broth at 35 °C. 0 day was the broth obtained from culture at 30 °C for 8 days. 4 days and 8 days represented the incubation time of the same broth in 35 °C and shaken at 180 rpm for 4 and 8 days, respectively.
